# Impact of incentives on COVID-19 vaccination; A systematic review

**DOI:** 10.3389/fmed.2022.810323

**Published:** 2022-09-08

**Authors:** Parham Mardi, Shirin Djalalinia, Reza Kargar, Mahnaz Jamee, Zahra Esmaeili Abdar, Mostafa Qorbani

**Affiliations:** ^1^Non-communicable Diseases Research Center, Alborz University of Medical Sciences, Karaj, Iran; ^2^Student Research Committee, Alborz University of Medical Sciences, Karaj, Iran; ^3^Development of Research and Technology Center, Deputy of Research and Technology, Ministry of Health and Medical Education, Tehran, Iran; ^4^Pediatric Nephrology Research Center, Research Institute for Children's Health, Shahid Beheshti University of Medical Sciences, Tehran, Iran

**Keywords:** COVID-19, vaccination, incentive, lottery, cash

## Abstract

**Introduction:**

Although vaccination is the most effective way to limit and overcome the COVID-19 pandemic, a considerable fraction of them are not intended to get vaccinated. This study aims to investigate the existing research evidence and evaluate the effectiveness and consequences of all incentives provided for increasing the uptake of COVID-19 vaccination.

**Methods:**

A systematic search in PubMed, Web of Science (WoS), and SCOPUS from 2020 until October 10, 2021, was conducted on experimental studies evaluating the effects of incentives including cash, lottery voucher, and persuasive messages on COVID-19 vaccination intention and uptake. The study selection process, data extraction, and quality assessment were conducted independently by two investigators using Consolidated Standards of Reporting Trials (CONSORT 2010) checklist.

**Results:**

Twenty-four records were included in the qualitative analysis. Most of the included studies assessed the effect of financial incentives. In 14 studies (58%) the assessed outcome was vaccination uptake and in nine (37.5%) others it was vaccination intention. One study considered self-reported vaccination status as the outcome. This study shows that high financial incentives and the Vax-a-million lottery are attributed to a higher vaccination rate, while the low amount of financial incentives, other lotteries, and persuasive messages have small or non-significant effects.

**Conclusion:**

Paying a considerable amount of cash and Vax-a-million lottery are attributed to a higher vaccination. Nevertheless, there is a controversy over the effect of other incentives including other lotteries, low amount of cash, and messages on vaccination. It is noteworthy that, inconsistency and imprecision of included studies should be considered.

## Introduction

Coronavirus Disease 2019 (COVID-19) is expanded worldwide, causing a global concern in all group ages (1–3). Vaccination is the most effective controllable measurement to limit and overcome the COVID-19 pandemic. It is estimated that in the best-case scenario using a vaccine with 90% efficacy against, herd immunity will be achieved by vaccinating at least 66% of the population (4), although some studies are more pessimistic predicting that herd immunity may not be achievable (5). The vaccination speed is currently limited in many countries due to insufficient production capacities and affordability (6). However, even in countries with adequate supplies and evidence for sufficient efficacy, vaccine hesitancy remains a non-negligible barrier in mitigating the COVID-19 pandemic (7).

Vaccine hesitancy is defined as the delay in acceptance or refusal of vaccination despite available vaccination services (8) and was introduced in 2019 by the World Health Organization (WHO) as one of the top ten threats to global health (9). The COVID-19 era may be associated with even more catastrophic consequences due to the rapid progression of the COVID-19 pandemic and the ongoing development of vaccine-resistant variants of SARS-CoV-2 (10, 11). The main reasons for vaccine hesitancy include concerns about vaccine side effects, distrust in their efficacy, misleading information about the vaccine's necessity and benefit, and conspiracy beliefs (7, 12). Also, vaccine hesitancy is linked to a lack of trust in COVID-19 vaccine safety and science (13).

Notably, the majority of vaccine-hesitant people are also highly resistant to required proof of vaccination. A previous study showed that a small of vaccine-hesitant people approve of requiring vaccination proof for access to international travel, indoor activities, employment, and public schools. To put it simply, not only are vaccine-hesitant individuals, not willing to get vaccinated themselves, but also they resist the rules that force others to get vaccinated. This leads to a block in improving COVID-19 vaccination coverage going forward and generates substantial challenges for ongoing vaccination campaigns to succeed (13, 14).

Both positive reinforcements [e.g., providing certain liberties to vaccinated people, monetary incentives (cash, lotteries, gift cards, free foods), facilitated access] (15) and negative reinforcements (restricted access to entertainment venues, not allowed to work) (12, 16) are applied to combat vaccine hesitancy and increase vaccination adherence. However, incentives (particularly financial ones) may, on the other hand, lead to a false interpretation of uptake-increasing strategies, further increasing society's resistance to vaccinations (17).

Besides incentives, other practical approaches for increasing vaccination willingness are proposed by different studies, including governmental transparency in providing information about vaccine details and decision making, altruistic messages (reflecting the responsibility of an individual to contribute to herd immunity), and highlighting the burden of losses associated with vaccine avoidance (18, 19). However, the degree to which these approaches can affect the rate of vaccination is not precise. The purpose of the present systematic review is to investigate the existing research evidence and evaluate the effectiveness and consequences of all types of incentives provided for increasing uptake/intention of COVID-19 vaccination.

## Methods

### Protocol

This study is a comprehensive systematic review of all available evidence on the association between incentives and COVID-19 vaccination. We followed a systematic review protocol that adheres to the PRISMA-P guidelines (20). All of the processes run based on details of the study protocol.

### Search strategy

The main root of search strategies developed is based on two main components of “incentives of vaccinations” and “COVID-19.” We searched the electronic data sources including PubMed, Web of Science (WoS), and SCOPUS based on the search strategy described in Supplementary Table 1. We also searched bioRxiv and medRxiv to include related preprint articles.

### Eligibility criteria

All relevant experimental studies [cross-sectional (CS), randomized trial (RT), factorial trial (FT), and quasi-experimental (QE)] have been included. All of the related review articles were evaluated for their references. There was no limitation in terms of the age and gender of the target groups and the time and language of the papers. All studies with duplicate citations were excluded. Moreover, other resources, related gray literature, publications' reference lists, and related key journals were searched for additional publications.

### Screening

The searched papers were exported to Endnote software. Initially, the relevancy of papers was evaluated based on their titles and abstracts, followed by a full-text assessment.

### Quality assessment

For remaining eligible studies, QA was conducted based on comprehensive recommended guidelines of the Consolidated Standards of Reporting Trials (CONSORT 2010) 25 item-checklist (21) by two independent research experts (SD and PM). Any disagreements resolve by another investigator (MQ).

### Data extraction

The data were extracted using a checklist recording bibliographic characteristics (citation, publication year, study year, provenance of study), general and methodologic characteristics (type of study, sample size, type of intervention, and type of outcome), and the main findings of each study. All search and data extraction process was done by two independent research experts (ZEA and RK).

### Ethical considerations

The present study was approved by the Ethical Committee of the Alborz University of Medical Sciences. All of the included studies are cited. Whenever we needed more information about a particular study, we contacted the corresponding author.

## Results

### Study selection process

**Figure 1:**
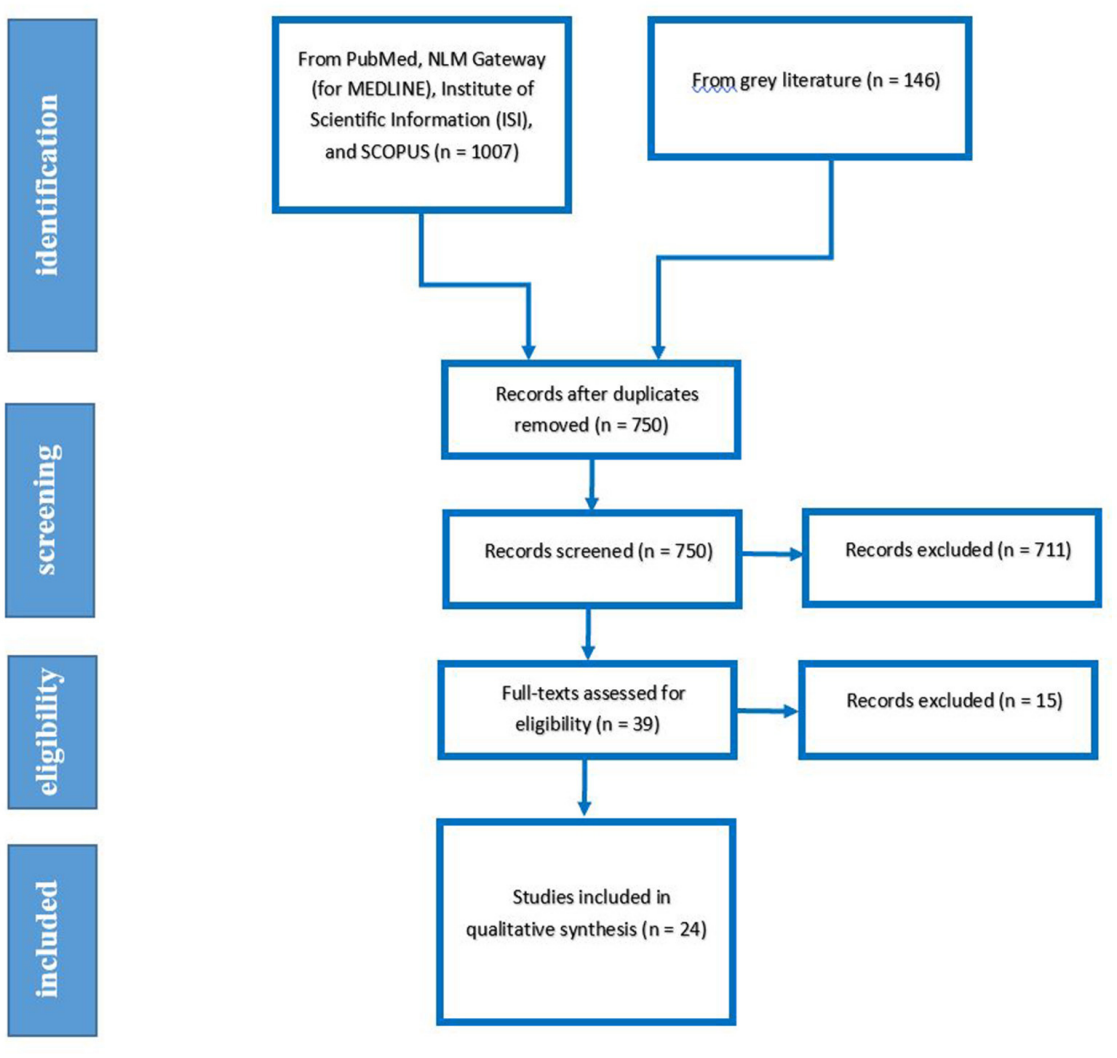
PRISMA flow diagram for study selection.

Our searches yielded 1,153 studies from previously mentioned databases and sources. After the rejection of duplicates, we screened 759 studies. We Excluded 601 and 110 papers after the title and abstract screening, respectively. After the full-text assessment, 24 studies were included in the qualitative synthesis. The detailed flow diagram is shown in Figure 1 (22–45).

### Studies and participants' characteristics

Among 24 included studies the design of 13 studies were QE, two studies were CS, six studies were RT and three studies were FT. Nine studies assessed the effect of financial incentives. In 14 studies (58%) the assessed outcome was vaccination uptake and in nine (37.5%) others it was vaccination intention. One study considered self-reported vaccination status as the outcome. Nineteen studies originated from the United States, followed by three papers that originated from Germany. The bibliographic and general characteristics of included studies are summarized in Table 1. Supplementary Table 2 provides details of the QA of included studies.

**Table 1:** Characteristics of included studies.

**Author, year (Reference)**	**Provenance**	**Sample size**	**Intervention**	**Study type**	**Outcome**	**QA (%)**
Acharya and Dhakal (22)	US	403,714	Vaccine lottery programs	CS	Self-reported Vaccination status	
Barber and West (23)	US (Ohio)	NR	Vax-a-million lottery	QE	Vaccine uptake	20/29 (68.96)
Brehm et al. (24)	US (Ohio and Indiana)	NR	Vax-a-million	QE	Vaccine uptake	20/29 (68.96)
Dai et al. (25)	US (North-Eastren)	93,354	Text message reminder	Two RTs	Vaccine uptake	17/27 (62.96)
Dave et al. (26)	US	NR	COVID-19 Vaccine Lottery Announcements	QE	Vaccine uptake	
Dutch et al. (27)	US	1,628	Video message	RT	Vaccine intention	22/29 (75.86)
Gandhi et al. (28)	US (Philadelphia)	NR	Vaccine regret lotteries	QE	Vaccine uptake	14/31 (45.16)
Jun and Scott (29)	Australia	2,375	Million Dollar Vax	CS	Vaccine uptake	16
Kachurka et al. (30)	Poland	5,931	Persuasive messages and financial incentives	RT	Vaccine intention	21/27 (77.77)
Kelkar et al. (31)	US (Florida)	264	A webinar	QE	Vaccine intention	20/33 (60.60)
Kerr et al. (32)	UK	2,097	Webpage message	QE	Vaccine intention	21/30 (70.00)
Kim (33)	US (Ohio)	NR	Lottery	QE	Vaccine uptake	15/24 (62.50)
Kluver et al. (34)	Germany	20,500	Freedom and financial incentives and vaccination at local doctor	FT	Vaccine uptake	20/33 (60.60)
Kreps et al. (35)	US	1,096	10$ and 100$ incentives	QE	Vaccine intention	22/27 (81.48)
Law et al. (36)	US (Ohio)	NR	Vax-a-million	QE	Vaccine uptake	
Mallow et al. (37)	US (Ohio)	213,288	Vax-a-million	QE	Vaccine uptake	21/29 (72.41)
Robertson et al. (38)	US	1,000	Financial incentive	RT	Vaccine uptake	21/30 (70.00)
Sehgal (39)	US (Ohio)	NR	Vax-a-million	QE	Vaccine uptake	17/23 (73.91)
Serra-Garcia and Szech (40)	US	1,040	Financial incentives	RT	Vaccine intention	16/26 (61.53)
Sprengholz et al. (41)	Germany	782	Legal and financial incentives	RT	Vaccine intention	21/31 (67.74)
Sprengholz et al. (42)	Germany	1,349	Communication and financial incentive	FT	Vaccine intention	22/31 (70.96)
Taber et al. (43)	US	274	Lottery and money	FT	Vaccine intention	21/30 (70.00)
Thirumurthy et al. (44)	US	NR	Financial incentives such as small guaranteed rewards and lottery token	QE	Vaccine uptake	
Walkey et al. (45)	US (Ohio)	NR	Vax-a-million	QE	Vaccine uptake	12/23 (52.17)

QA, quality assessment; RT, randomized trial; QE, quasi experimental; FT, factorial trial; NR, not reported; CS, cross-sectional.

### Qualitative synthesis

Table 2 not merely summarizes the effects of different incentives on indices related to vaccine uptake and intention but also shows how the surge in vaccine uptake as a result of incentives can prevent COVID-19 infection and its outcomes.

**Table 2:** Qualitative analysis of included studies.

**Author, year**	**Provenance**	**Sample size**	**Intervention**	**Outcome**	**Methods**	**Findings**
Acharya and Dhakal (22)	US	40,3714	Vaccine lottery programs	Self-reported vaccination status	Using Household Pulse Survey (HPS), 11 states implementing a vaccine lottery program and 28 states with no such program were compared based on a difference-in-difference (DiD) analysis.	•Augmented synthetic control (ASC) analysis revealed that lottery programs were associated with 23.12% (0.208 log points) increment in the new daily vaccination rate. •Vaccine lottery programs were helpful in Ohio, Maryland, Oregon, New Mexico, New York and Washington but not in Arkansas, Kentucky, California, Colorado, and West Virginia. •Although overally vaccine lottery programs increases vaccination rate, findings differ across states.
Barber and West (23)	US (Ohio)	NR	Vax-a-million	Increase in the first dose vaccinated share of Ohio population/COVID-19 cases/ICU admissions due to COVID-19	Comparing Ohio with a synthetic control from May 12 to June 20	•The first dose vaccinated share of the Ohio population was increased by 1.49% [95%CI 1.12 to 1.81]. •The change in the cumulative total number of COVID-19 cases was−24.51 per 100,000 persons [95%CI −36.92 to −12.25] until June 20, and it was −125.4 per 100,000 people [95%CI −137.6 to −112.9] until July 18. •Change in the cumulative total number of ICU admissions due to COVID-19 was −8.238 [95%CI −10.44 to −6.06] per 100,000 until June 20, and it was −41.40 [95%CI −43.52 to −39.15] until July 18.
Brehm and Brehm (24)	US (Ohio and Indiana)	NR	Vax-a-million	Number of vaccinated people/vaccination rate	Comparing counties in Ohio border with Indiana with counties in Indiana border with Ohio	•The increase in vaccination rate at the first week of the lottery was 6.3 doses per 10,000. •The difference in vaccination rate at the second week of the lottery was 3.5 doses per 10,000. •The additional number of people who get vaccinated was 80,807 people.
					Comparing Ohio with a synthetic control	•Compared to synthetic control of Ohio (in counties with over 250,000 people), the vaccination rate was increased by 64 vaccinated people 10,000 participants. •Compared to synthetic control of Ohio (in counties with <250,000 people), the vaccination rate was increased by 37 vaccinated people 10,000 participants. •The additional number of people who get vaccinated in Ohio compared to the synthetic control was 77,000.
Dai et al. (25)	US (North-Eastern)	93,354	Text message reminder	To get vaccinated	Comparing people who received a text with people who did not receive	•The increase in the vaccination rate due to the first reminder was 3.57%. •The increase in the vaccination rate due to the second reminder was 1.06%.
					Comparing different content of each reminder	•13.89% of participants in the control group get vaccinated. •17.13% of participants in the basic reminder group get vaccinated. •18.22% of participants in the ownership reminder group get vaccinated. •16.90% of participants in the Basic video group get vaccinated. •18.16% of participants in ownership with the video group get vaccinated.
Dave et al. (26)	US	NR	COVID-19 Vaccine Lottery Announcements	Vaccination rate	A difference-in-differences framework was used for the analysis, which compared daily reported COVID-19 vaccinations per 1,000 population before and after the lottery announcement	•No statistically significant association was detected between a cash-drawing announcement and the number of vaccinations before or after the announcement date. •Estimates of the association between a lottery announcement and vaccination rates were statistically insignifcant. •Lottery based incentiviation is less effective than incentives that pay with certainty.
Dutch et al. (27)	US	1,628	Video message	Wanting information about where to get vaccinated	Survey	•16% Percent of people willing to get information about where to get vaccinated after watching a video regarding the health benefits of vaccination (OR = 1). •14% percent of people are willing to get information about where to get vaccinated after watching a video regarding lottery [OR = 0.82 (95%CI 0.57–1.17)]. •22% percent of people are willing to get information about where to get vaccinated after watching a video regarding cash vouchers [OR = 1.53 (95%CI 1.11–2.11)].
Gandhi et al. (28)	US (Pennsylvania)	3,827,656	Three high-payoff vaccine regret lotteries	Number of first-dose vaccinations	Comparing Philadelphia county (lottery) with adjacent counties (no lottery)	•383 [−52, 819] extra vaccinations per 100,000 people were done in Philadelphia county (lottery) compared to adjacent counties (no lottery). •Limited evidence of impact on vaccine uptake even with increased odds of the lottery was evident.
Jun and Scott (29)	Australia	2,375	Million Dollar Vax	Proportion of respondents who had any vaccination	Taking the Pulse of the Nation Survey	•Overall, participants who entered in to the competition were 2.27 times more likely to get vaccinated after the initiation of the competition. •Although the intervention did not significanty influence the number of participants who get the first dose of vaccine, it was correlated with number of participants who get the second dose.
Kachurka et al. (30)	Poland	5,931	Persuasive messages and financial incentives	Vaccination attitudes	Nation-wide online experiment	•No incentives such as persuasive messages and paying money reduces the vaccine hesitancy. •45 % of participants in this study were unwilling to get vaccinated regardless of the type of incentive.
Kelkar et al. (31)	US (Florida)	264	A webinar (cancer in the times of coronavirus COVID-19 vaccine)	Percentage of people intended to receive the vaccine	A survey in patients diagnosed with cancer	•71% and 82.5% of people were intended before and after the webinar, respectively. •24.0% and 15.4 % of people were unsure before and after the webinar, respectively. •5% and 2% of people were not intended before and after the webinar, respectively.
					*T-*test regarding perspectives on vaccine	•Participation in the webinar leads to an enhancement in patients perspectives in terms of (*p* < 0.05): 1. Vaccine effectiveness 2. Vaccine safety 3. Information about how to get vaccinated 4. Enthusiasm to encourage friends and family 5. Getting out of the way to get vaccinated
Kerr et al. (32)	UK	2,097	Webpage message comprising fact box, Q/A, Approval, and mechanism of how vaccines induce immunity messages	Intention to get vaccinate	Survey	•None of the fact boxes, Q/A, Approval, and mechanism of how vaccines induce immunity messages increased Intention to get vaccinated compared to the control group (*p* > 0.05). •People who receive Fact box and Q/A messages feel significantly more informed than the control group (*p* < 0.05). •None of the fact boxes, Q/A, Approval, and mechanism of how vaccines induce immunity messages increased the rate of receiving vaccine if offered compared to the control group (*p* > 0.05).
				Feeling informed		
				Receiving vaccine if offered		
Kim (33)	US (Ohio)	NR	Lottery, unconditional version of the Lottery and Transfer^a^	Participation rate	Experimental comparison	•In both Lottery and UnconLottery, 74% of subjects participated. •Participation rates were significantly higher in lottery group compared to control group (t-stat= 1.9802, *p* = 0.0499). •Participation rates were significantly higher in lottery group compared to transfer group (t-stat = 2.714, *p* = 0.0076).
Kluver et al. (34)	Germany	20,500	Additional freedom, financial freedom, getting vaccinated at the local doctor	Vaccine uptake	Factorial survey experiment	•25€ incentive increases the vaccine uptake by 1 pp. •50€ incentive increases the vaccine uptake by 2.2 pp. •Additional freedom incentive increases the uptake by 2.5 pp. •Getting vaccinated at the local doctor incentive increases the uptake by 3pp. •All incentives together incentive increase the uptake by 13pp.
Kreps et al. (35)	US	1,096	10$ and 100$ incentives	Would choose to get vaccinated or not	Survey	•The effect of 10$ incentive on vaccine intention was not significant (Regression coefficient = 0.01, SE = 0.01). •The effect of 100$ incentive on vaccine intention was not significant (Regression coefficient = 0.00, SE = 0.01). •Paying 20$ significantly decreased vaccine intention (Regression coefficient = −0.04, SE = 0.01).
Law et al. (36)	US	NR	Vax-a-million	Vaccination rate	Using interrupted time series analyses with segmented regression in a data obtained from the US Centers for Disease Control and Prevention	•The vaccination rate decreased before lottery announcements by 2.8 vaccinations/100 000 people/day. •Vaccine administrations did not significantly increase [−0.4 (95% CI, −23.5 to 22.7) vaccinations/100 000 people] after announcement of lottery. Moreover, vaccination trends did not significantly change compared to prelottery trends. •This study revealed that vaccine lottery incentive programs in the US were not associated with significantly increased rates of vaccinations.
Mallow et al. (37)	US (Ohio)	213,288	Vax-a-million	Number of COVID-19 vaccines per day per low income county	Difference-in-difference (DiD)	•The average number of COVID-19 vaccines per day was 140.44 [95%CI 133.37–147.89] per day per low-income county before the lottery, while after the lottery, this number increased to 165.92 [95%CI 147.80–186.26]. •The increase of vaccinations due to lottery was 25.48 [95%CI 14.43–38.37] per low-income county per day.
Robertson et al. (38)	US	1,000	Financial incentive	Increase in Vaccine uptake	Online survey experiment	•1,000$ incentive increases the vaccine uptake by 7.6pp (significant at the 90% level). •1,500$ incentive increases the vaccine uptake by 11.7pp (significant at the 99% level). •2,000$ incentive increases the vaccine uptake by 4.1pp (not significant). •Overall, offering incentives leads to an 8 pp increase in people who say “yes” to the vaccine and a 6 pp decrease in participants who respond “no.”
Sehgal (39)	US (Ohio)	NR	Vax-a-million	Number of vaccinated people / vaccination rate	Comparing Ohio with a synthetic control comprised of 11 states.	•The percentage of people with the first dose was increased by 0.98% [95%CI 0.42–1.54%] compared to synthetic control of Ohio.
Serra-Garcia and Szech (40)	US	1,040	Financial incentives	Wanted to receive the vaccine	Online experiment	•20$ incentive changes the vaccine intention by −5 pp [95%CI −6.7– −3]. •100$ incentive increases the vaccine intention by 4.5pp (95%CI or p-value was not reported). •500$ incentive increases the vaccine intention by 13.6pp (*p* < 0.001). •The opt-out condition increases the vaccine intention by 6.8 pp [95%CI 1.2, 12.4].
Sprengholz et al. (41)	Germany	782	Legal and financial incentives	Vaccine intentions (VI)	Survey	•Vaccine intention was 65.1% in the group with Legal privileges, while it was 61.4% in the group without legal privileges (*p* = 0.300). •For monetary incentive, the significance level reached 0.05 when 3,250 euros were offered. •Ten thousand euros increased the vaccine intention by 10.4% compared to the 0€ group. •143 out of 782 participants were willing to vaccinate only when the financial incentive was offered.
Sprengholz et al. (41)	Germany	1,349	Communication^b^ and financial incentive (25euro to 200euro)	How likely participants were to get vaccinated if they had the chance to do so in the next month	Survey Regression analysis (the outcome was based on a 7-point scale)	•Communication^2^ was not significantly associated with vaccine intention [β = −0.34 (95%CI −0.84–0.15), SE = 0.25]. •Payment was not significantly associated with vaccine intention (β = 0.22 [95%CI −0.19–0.62], SE = 0.25). •The cumulative effect of payment and Communication^b^ was not significantly associated with vaccine intention [β = −0.05 (95%CI −0.62–0.52) SE = 0.29].
Taber et al. (43)	US	274	Lottery and money	Vaccine intention	Survey (online experiments)	•In 37.2% of participants, the lottery was an intention to get vaccinated (5 people win 1,000,000$ was significantly more favorable). •10.9 percent of the participants would vaccinate with getting <10$ as an incentive. •15.6 percent of the participants would vaccinate with getting 11$-100$ as an incentive. •20.4 percent of the participants would vaccinate with getting 101$-2000$ as an incentive. •22.2 percent of the participants would vaccinate with getting more than 2,000$ as an incentive. •26.3% of participants would not vaccinate either for a guaranteed amount of money or lottery.
Thirumurthy et al. (44)	US	NR	Financial incentives such as small guaranteed rewards and lottery token	Vaccine administrations	Difference-in-differences analyses and combination of information on statewide incentive programs in the US with data on daily vaccine doses administered in each state	•Statewide programs were not significantly correlated with a change in vaccination uptake. •No significant difference was evident in vaccination trends among states with and without financial incentives. •Their findings were verified with heterogeneity analyses, which indicated that neither lotteries nor guaranteed rewards were associated with a significant change in vaccination rates.
Walkey et al. (45)	US (Ohio)	NR	Vax-a-million	Vaccination rate trend	Interrupted time-series study	•After the introduction of the Ohio vaccine lottery, the declines in daily vaccination rates slowed in Ohio. The change from before the lottery introduction was 6 [95%CI 0–11] per 100 000 people (*p* = 0.05). •After introducing the Ohio vaccine lottery, the difference between the US and Ohio was −12 [95%CI −46–22] per 100 000 people (*p* = 0.51).

^a^Transfer: Every subject who chose [P] receives an equal split of 250 additional points when 20 or more subjects choose [P].

^b^Highlighting the effects of individual vaccination on infections and herd immunity.

NR, not reported.

Thirteen of included studies revealed the effects of the lottery on vaccination uptake or intention. Vax-a-million lottery in Ohio, United States, was attributed to a significant increase ranging from 0.98% (39) to 1.49% (23) in the percentage of the first dose vaccinated people compared to synthetic control. This intervention not only slowed the decline in vaccination rate (37) but also led to the vaccination of additional 77,000 Ohioans compared to synthetic control (24). Nevertheless, some other studies revealed insignificant or negligible findings regarding the lotteries in the US (36). Included studies also revealed contradictory findings for other lotteries. While most studies showed limited or no evidence of impact (22, 26, 28, 29, 44), others demonstrated that lottery should be considered as a motivation for vaccination in a significant fraction of participants (33, 43).

Similarly, most studies revealed non-significant effects of persuasive messages and communication in incrementing the general population's intention to get vaccinated (21, 23, 31). Nevertheless, this intervention significantly enhances the intentions and perspectives of patients diagnosed with cancer (31). It should be noted that text messages and first reminders lead to a higher vaccination intention than video messages and second reminders, respectively (25).

When paid in high amounts, financial incentives significantly increase the participants' intention to vaccination (40, 42, 43). Conversely, studies included in this review showed that not merely the effects of the low amount of financial incentives are non-significant (30), but also that these incentives can lead to a decreased vaccination intention (40).

## Discussion

The present study was performed to systematically review the effect of some interventions, such as lotteries, persuasive messages, and financial incentives, and a series of other variables such as legal incentives on getting vaccinated or people's intention to get vaccinated. The lottery has driven minor changes in vaccination share among people. Messages' effects are generally non-significant; however, they may be beneficial in a particular group of people. Conversely, most studies have shown that financial incentives can effectively increase the vaccination rate.

Various incentives and behavioral nudges have been applied to abrogate vaccine hesitancy and increase vaccination coverage. On May 13, 2021, Ohio announced the Vax-a-Million, a free weekly lottery, which ran from May 26 to June 23 for Ohioans who had received at least one COVID-19 vaccine. By June 20, the end of the lottery registration period, nearly 3.5 million adults and 155,000 children had registered for the free lottery (39). Several studies have examined the effect of this incentive program. Overall, the results showed that the first dose vaccinated share of the population, vaccination rate, and the number of vaccinated people increased. Furthermore, the cumulative total number of COVID-19 cases and the total number of ICU admissions due to COVID-19 decreased after the beginning of the lottery. As mentioned earlier, these changes have been enough to be considered meaningful, although evidence on lotteries other than Vax-a-Million is limited (28).

By considering the additional number of people who have been encouraged to get vaccinated because of the Ohio Vax-a-million, Barber and West (23) showed that the cost of the Vax-a-million scheme was less than one-tenth of the potential costs incurred in the absence of the lottery, which indicates the value of such schemes. Furthermore, recent evidence suggests that a lottery is more cost-effective than a lump-sum transfer payment (33). The budget allocated to Ohio's Vax-a-million was $75 for each additional injected dose, much more cost-effective than paying to everyone.

In contrast to lotteries, text message reminders for vaccination do not seem to considerably affect vaccine hesitancy. Although a slight increment in the vaccination rate was observed after the first message reminder, the second text message reminder had a meaningless influence. It is worth noting that video messages had a more negligible effect on the vaccination rate than text messages (25). On the other hand, messages regarding cash vouchers raised people's willingness to get information about vaccination; however, it is unclear how much this intervention will actually lead to getting vaccinated (27).

Unlike the general population, webinars on cancer and COVID-19 vaccination represented promising results. The percentage of patients with cancer who intended to receive the vaccine increased, and patients' perspectives in terms of vaccine effectiveness, vaccine safety, information about how to get vaccinated, enthusiasm to encourage friends and family, and getting out of the way to get vaccinated were notably improved (31). Therefore, measurements to increase awareness among target populations with special conditions can be taken to address their doubts about vaccination.

Among the interventions we reviewed, financial incentives (paying cash) generally had higher levels of significance. However, determining the amount of money paid should be considered thoroughly because it has been shown that small amounts of money as an incentive can be ineffective or even have reverse effects (35, 40). In other words, some studies demonstrated that offering little payment often signals to individuals that a particular behavior is unpredictable, hazardous, or risky, which may in turn lead to lower vaccination uptake (46). But paying higher amounts, such as 1,000 or $1,500, has significant effects on people's willingness to get vaccinated (38, 40). It can be conjectured that economically vulnerable populations are more likely to get vaccinated by financial incentives. Nonetheless, other factors such as young age, psychological antecedent (less confidence about vaccine safety, more complacency about no necessity of vaccination) (42), and belonging to specific political groups (democrats and independents vs. republicans) (38) seem to contribute to the higher responsiveness to monetary incentives. It is important to note that the correlation between the size of incentive and vaccine uptake is not linear, as one study showed that huge financial incentives ($2,000) appear to be less effective than moderately sized incentives ($1,500) and even maybe counter-productive is some ethnic groups (Black and Latino Americans) (38). This finding may somehow decrease concerns regarding the possibility of disturbing autonomous vaccination decisions in those of limited means.

There is controversy over whether it is ethical to encourage people to get vaccinated through financial incentives. Some experts believe that paying cash and giving lottery vouchers undermines the moral spirit of performing tasks such as maintaining and promoting the personal community's public health and contributing to the mitigation of pandemics. In addition, they state that many people are willing to get vaccinated even without the need to receive money, which leads to the unnecessary waste of substantial financial resources (46).

Conversely, other studies point to the history of such schemes, arguing that these incentives can reduce the burden of COVID-19 in general. Also, upgrading and increasing vaccination can provide better safety for disadvantaged individuals (47, 48). In addition, these incentives create a double motivation. They can at least make people get vaccinated in a reduced timetable, which eventually leads to decreased number of infected patients and declined mortality rate (17, 39). To sum up, similar to Brewer et al. research, our findings suggest that vaccine incentives are more effective when they are delivered immediately, recipients value them, and more importantly, their receipt is certain (49).

### Limitations

This study had several limitations; First, the inclusion criteria and experimental setting of the studies we have mentioned for incentives may not cover a wide range of people, and this limitation may change the results of this study in reality. Second, Most studies reviewed in this paper originated from the United States and Europe. Prior to extending the results of this study to other countries, considerations should be taken into account according to the demographic, cultural, and structural features. Third, most of the included experimental studies suffered from a lack of a well-defined control group which may have affected the results. Fourth, the findings of this study could be affected by, inconsistency and imprecision of included studies.

### Implications

It is estimated that the threshold of 60–70% of the population gaining immunity is essential to achieve COVID-19 herd immunity (4). As a result of high vaccine hesitancy, this threshold is probably impossible to achieve without vaccine incentives. This study assists policymakers around the globe to opt for the most effective incentive to boost COVID-19 vaccination in their countries. Governments should keep in mind that although vaccination incentives were introduced as practical tools in accelerating the vaccination, more experimental studies in variable geographical regions and different ethnic groups concerning each country's specifications are still needed.

### Conclusion

This review showed that participants were more likely to get vaccinated when incentivized by a high amount of cash. Moreover, while Vax-a-million significantly increases the uptake of the COVID-19 vaccine, the effects of other lotteries and persuasive messages were non-significant or marginally significant.

## Data availability statement

The raw data supporting the conclusions of this article will be made available by the authors, without undue reservation.

## Author contributions

RK and ZE: data extraction. PM, SD, and MQ: systematic search and quality assessment. PM: drafting. MQ and MJ: revision. MQ: supervision. All authors read and approved final version of manuscript.

## Conflict of interest

The authors declare that the research was conducted in the absence of any commercial or financial relationships that could be construed as a potential conflict of interest.

## Publisher's note

All claims expressed in this article are solely those of the authors and do not necessarily represent those of their affiliated organizations, or those of the publisher, the editors and the reviewers. Any product that may be evaluated in this article, or claim that may be made by its manufacturer, is not guaranteed or endorsed by the publisher.
