# Supplementary material for: Impact of incentives on COVID-19 vaccination; A systematic review
Source: Front Med (Lausanne). 2022 Sep 8;9:810323. doi: 10.3389/fmed.2022.810323 (PMC9492889; doi:10.3389/fmed.2022.810323)
Supplement: Supplementary file 1 [file Table_1.DOCX]

| **Supplementary table 1. Search strategy** |
| --- |
| **Pubmed** |
| ((("COVID-19"[Mesh]) OR ((((("COVID 19"[Title/Abstract]) OR ("COVID-19"[Title/Abstract])) OR ("nCoV Infection"[Title/Abstract])) OR ("Coronavirus Disease"[Title/Abstract])) OR ("SARS CoV 2"[Title/Abstract]))) AND ((((Vaccination[MeSH Major Topic]) OR (Vaccination[MeSH Terms])) OR (Immunization[Title/Abstract])) OR (vaccin*[Title/Abstract]))) AND (((((((((((incentive[Title/Abstract]) OR (Lottery[Title/Abstract])) OR (Paying[Title/Abstract])) OR (Cash[Title/Abstract])) OR (encourag*[Title/Abstract])) OR (Prize[Title/Abstract])) OR (Award[Title/Abstract])) OR (Gift[Title/Abstract])) OR ("Livelihood pack"[Title/Abstract])) OR (Discount[Title/Abstract])) OR ("Financial pack"[Title/Abstract])) |
| **Scopus** |
| ( ( ( TITLE-ABS-KEY ( *"COVID-19"* )  OR  TITLE-ABS-KEY ( *"COVID 19"* )  OR  TITLE-ABS-KEY ( *"nCoV Infection"* )  OR  TITLE-ABS-KEY ( *"Coronavirus Disease"* )  OR  TITLE-ABS-KEY ( *"SARS CoV 2"* ) ) )  AND  ( ( TITLE-ABS-KEY ( *vaccin** )  OR  TITLE-ABS-KEY ( *immunization* ) ) ) )  AND  ( ( TITLE-ABS-KEY ( *incentive* )  OR  TITLE-ABS-KEY ( *lottery* )  OR  TITLE-ABS-KEY ( *paying* )  OR  TITLE-ABS-KEY ( *prize* )  OR  TITLE-ABS-KEY ( *cash* )  OR  TITLE-ABS-KEY ( *award* )  OR  TITLE-ABS-KEY ( *gift* )  OR  TITLE-ABS-KEY ( *"Livelihood pack"* )  OR  TITLE-ABS-KEY ( *discount* )  OR  TITLE-ABS-KEY ( *"Financial pack"* )  OR  TITLE-ABS-KEY ( *encourag** ) ) )  AND  ( EXCLUDE ( DOCTYPE ,  *"ch"* )  OR  EXCLUDE ( DOCTYPE ,  *"er"* ) ) |
| **ISI/WOS** |
| TOPIC: (COVID-19) OR TOPIC: ("COVID 19") OR TOPIC: ("nCoV Infection") OR TOPIC: ("Coronavirus Disease") OR TOPIC: ("SARS CoV 2")  TOPIC: (vaccin*) OR TOPIC: (immunization)  Indexes=SCI-EXPANDED, SSCI, A&HCI, CPCI-S, CPCI-SSH, BKCI-S, BKCI-SSH, ESCI Timespan=All years TOPIC: (incentive) OR TOPIC: (lottery) OR TOPIC: (paying) OR TOPIC: (prize) OR TOPIC: (cash) OR TOPIC: (award) OR TOPIC: (gift) OR TOPIC: ("Livelihood pack") OR TOPIC: ("Financial pack") OR TOPIC: (discount) OR TOPIC: (encourag*)  Indexes=SCI-EXPANDED, SSCI, A&HCI, CPCI-S, CPCI-SSH, BKCI-S, BKCI-SSH, ESCI Timespan=All years  Indexes=SCI-EXPANDED, SSCI, A&HCI, CPCI-S, CPCI-SSH, BKCI-S, BKCI-SSH, ESCI Timespan=All years |
